# Mild Mitochondrial Uncoupling and Calorie Restriction Increase Fasting eNOS, Akt and Mitochondrial Biogenesis

**DOI:** 10.1371/journal.pone.0018433

**Published:** 2011-03-31

**Authors:** Fernanda M. Cerqueira, Francisco R. M. Laurindo, Alicia J. Kowaltowski

**Affiliations:** 1 Departamento de Bioquímica, Instituto de Química, Universidade de São Paulo, São Paulo, São Paulo, Brazil; 2 Instituto do Coração, Faculdade de Medicina, Universidade de São Paulo, São Paulo, São Paulo, Brazil; Paris Institute of Technology for Life, Food and Environmental Sciences, France

## Abstract

Enhanced mitochondrial biogenesis promoted by eNOS activation is believed to play a central role in the beneficial effects of calorie restriction (CR). Since treatment of mice with dinitrophenol (DNP) promotes health and lifespan benefits similar to those observed in CR, we hypothesized that it could also impact biogenesis. We found that DNP and CR increase citrate synthase activity, PGC-1α, cytochrome c oxidase and mitofusin-2 expression, as well as fasting plasma levels of NO^•^ products. In addition, eNOS and Akt phosphorylation in skeletal muscle and visceral adipose tissue was activated in fasting CR and DNP animals. Overall, our results indicate that systemic mild uncoupling activates eNOS and Akt-dependent pathways leading to mitochondrial biogenesis.

## Introduction

Mitochondrial respiratory activity is a recognized factor in lifespan: Increments in respiratory activity enhance cell and animal longevity [1]–[6]. Respiratory activity can be increased through different strategies including enhanced availability of substrates, mitochondrial uncoupling or augmented activity of electron transport enzymes resulting from changes in mitochondrial biogenesis and dynamics [7]–[11]. The specific effects of each of these interventions on lifespan remain to be fully uncovered.

Speakman *et al.* [12] elegantly demonstrated that mice with increased respiratory rates and reduced energetic conversion efficiency due to spontaneously uncoupled mitochondria lived longer than their counterparts. Indeed, different uncoupling strategies were able to extend lifespan in models ranging from yeast to mammals [1]–[4], [13]–[15]. Furthermore, knockout mice for uncoupling protein 2 present shortened lifespans [16]. These findings are in line with the “uncoupling to survive” hypothesis proposed by Brand [6], suggesting that uncoupling could be an approach to promote lifespan extension due to its ability to prevent the formation of reactive oxygen species (ROS). Indeed, mild mitochondrial uncoupling is a highly effective intervention to prevent the formation of ROS [9], [17].

Interestingly, increased respiratory rates associated with mitochondrial uncoupling are measured in some organisms under caloric restriction (CR) [1], [18], the most widely reproduced approach associated with lifespan extension [19]–[20]. CR-induced changes in respiratory activity result in lower mitochondrial ROS generation and oxidative damage [18], [21]–[22] reinforcing the “uncoupling to survive” hypothesis [6], [12].

CR also increases the number of functional respiratory units (mitochondrial biogenesis [23]–[25]) and promotes changes in mitochondrial dynamics which may affect respiratory rates [25]. Nisoli *et al.* [8]; [26], [27] demonstrated that mitochondrial biogenesis was essential for many beneficial effects of dietary limitation in mice, and that this process was driven by nitric oxide (NO^•^) signaling [26]–[29]. Biogenesis-promoting NO^•^ is synthesized by the endothelial isoform of nitric oxide synthase (eNOS), an enzyme present in diverse tissues and sensitive to nutritional status which modulates Akt activity and, consequently, eNOS phosphorylation [30]. In addition, long term exposure to elevated ROS levels impairs eNOS activity [31]–[32]. As a result, mitochondrial biogenesis is regulated by changes in animal energy metabolism as well as the redox state of the tissue.

We recently demonstrated that murine lifespan can be extended by low doses of the mitochondrial uncoupler 2,4-dinitrophenol (DNP) in a manner accompanied by weight loss, lower serological levels of glucose, insulin and triglycerides as well as a strong decrease in biomarkers of oxidative damage and tissue ROS release [4]. Similar effects have been repeatedly reported using CR diets (reviewed in [33]). Based on the similarities between these two interventions, we hypothesized that DNP treatment could also lead to enhanced mitochondrial biogenesis.

In this manuscript, we measured the effects of DNP treatment and CR on mitochondrial biogenesis and associated pathways. We observed that both DNP and CR increase mitochondrial biogenesis as well as basal Akt and eNOS activities, confirming that signaling events in both treatments converge. This is the first experimental evidence that uncoupling *in vivo* can impact mitochondrial number and function.

## Materials and Methods

### Animals

All experiments were conducted in strict agreement with the National Institutes of Health Guidelines for Humane Treatment of Animals and were reviewed and approved by the local Animal Care and Use Committee (*Comissão de Ética em Cuidados e Uso Animal*). Female, 4-week-old Swiss mice were separated in 3 groups: **AL**, fed *ad libitum* with an AIN-93-M diet prepared by Rhoster (Campinas, SP, Brazil); **DNP**, fed *ad libitum* with the same diet and treated with the mitochondrial uncoupler DNP added to the drinking water at a final concentration of 1 mg·L^−1^; **CR**, fed with 60% of the same diet supplemented with micronutrients to reach the vitamin and mineral levels consumed by AL animals [33]. Body mass and food consumption were recorded weekly. CR feedings were adjusted weekly by weight based on AL food consumption measured one week prior. Food was offered to CR mice every day at 6 pm. The animals were lodged 5 individuals per cage and given water *ad libitum*. After 6 months of dietary intervention or DNP treatment, mice were sacrificed after 12 hours fasting or after 12 hours fasting followed by *ad libitum* feeding (post-prandial experiments).

### Citrate synthase activity

Visceral adipose tissue and skeletal muscle samples were homogenized using an electric potter in lysis buffer (50 mM sodium phosphate, pH 7.4, 10% glycerol, 1% octyl phenol ethoxylate, 10 mM sodium orthovanadate, 10 mM sodium fluoride, 10 mM sodium pyrophosphate, supplemented with Sigma protease inhibitor mixture). After 30 min over ice, tissues lysates were centrifuged (13,000 *g*, 20 min, 4°C), and the resulting supernatants were collected. A reaction mixture of 20 mM Tris-HCl, pH 8.0, 0.42 mM acetyl-coenzyme A, 0.1 mM DTNB and 20 µg of total protein was incubated at 37°C for 5 min. The reaction was initiated by the addition of 0.5 mM oxaloacetate. The reduction of 5′,5′-dithiobis(2-nitrobenzoic acid) by citrate synthase was measured spectrophotometrically during 5 min at 412 nm (extinction coefficient = 13.6 mM^−1^·cm^−1^ [34]). Activities are expressed as nmol of citrate·min^−1^·mg^−1^.

### Plasma nitrite levels

Blood was collected by cardiac puncture from fasted mice (12 hours) immediately after sacrifice. Heparinized blood samples were centrifuged at 2,000 rpm for 5 min and plasma stored at −80°C until analysis. Levels of nitrite (NO_2_^−^), a marker of NO^•^ levels, were measured through chemiluminescence of the reaction between ozone and NO^•^ generated by the reduction of the sample with vanadium chloride in acid at 95°C, using an NO^•^ analyzer (Model 208A; Sievers Instruments Inc., Boulder, CO, USA) according to the manufacturer protocols [35]–[36].

### Western Blots

Total proteins from tissue lysates were diluted in Laemmli sample buffer (100 mM Tris.HCl, 2% w/v SDS, 10% v/v glycerol, 0.1% bromophenol blue) containing 100 mM dithiothreitol, with exception of eNOS and phospho-eNOS Western Blots, which were performed without the reducing agent. After heating at 90°C for 5 min, proteins were separated by SDS-PAGE and transferred onto nitrocellulose membranes. After membranes were blocked with 5% BSA, the detection of individual proteins was carried out by blotting with specific primary antibodies against cytochrome c oxidase (Sigma, 1∶2,000), PGC1-alpha (Cell Signaling, clone 3G6, 1∶1,000), mitofusin-2 (Cell Signaling, 1∶2,000), eNOS (Sigma, 1∶3,000), phospho-eNOS^Ser1177^ (Cell Signaling, C9C3 clone, 1∶1,000), Akt (Calbiochem, 1∶1,000), phospho-Akt^Ser473^(Cell Signaling, 1∶3,000) and gamma-actin (Sigma, 1∶2,000). Chemiluminescent detection using a secondary peroxidase-linked anti-rabbit (Calbiochem, 1∶10,000) or anti-sheep IgG (Calbiochem, 1∶13,000) and a detection system from Pierce KLP (Rockford, IL, USA) was performed. Signals were quantified by densitometry using ImageQuant® (Amersham Biosciences) and corrected using beta-actin.

## Results and Discussion

### DNP and CR increase mitochondrial biogenesis

**Figure 1:**
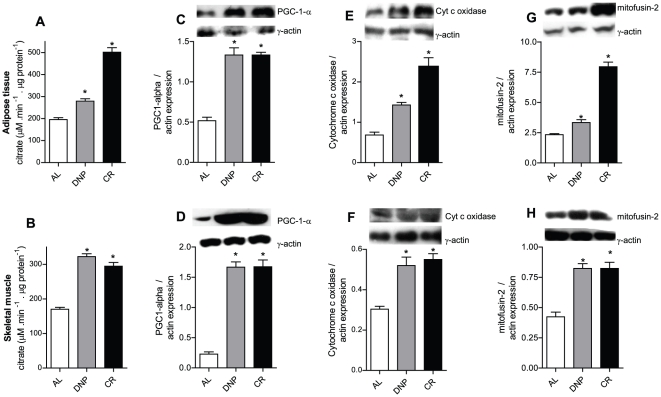
CR and DNP promote mitochondrial biogenesis. Citrate synthase activity was determined spectrophotometrically in visceral adipose tissue (A) and skeletal muscle (B) homogenates. Mitochondrial biogenesis markers were quantified using Western Blots: PGC1-alpha, visceral adipose tissue (C) and skeletal muscle (D); cytochrome c oxidase, visceral adipose tissue (E) and skeletal muscle (F); mitofusin-2, visceral adipose tissue (G) and skeletal muscle (H). γ-actin was used as a loading control. Typical blots are shown above average densitometry results. AL, *ad libitum*; DNP, dinitrophenol-treated mice; CR, calorically-restricted mice. *p<0.05 vs. AL.

Dietary restriction has previously been shown to enhance mitochondrial biogenesis [23]–[25]. We were able to reproduce this finding under our experimental conditions, as indicated by the fact that citrate synthase activity, PGC1-alpha and cytochrome c oxidase expression, markers for mitochondrial biogenesis [24]; [27]; [36]–[38], increased in the adipose tissue and skeletal muscle of CR animals relative to AL (Figs. 1A–1F). Interestingly, DNP-treated animals also presented significant increments in these biomarkers in both tissues, indicating that systemic mild uncoupling promotes mitochondrial biogenesis. Further confirming that both DNP and CR enhance mitochondrial content, we found that the expression of mitofusin-2, a mitochondrial protein involved in the control of morphology and dynamics [37]; [38], was also strongly increased by CR and DNP in both visceral adipose tissue and skeletal muscle (Figs. 1G and 1H, respectively). Interestingly, since mitofusin-2 is involved in the control of mitochondrial morphology and dynamics, our finding suggests that these characteristics are involved in the beneficial effects of these treatments. In this line, van Diepeningen *et al.* [25] recently found evidence that changes in mitochondrial morphology and dynamics occur in a CR model in *Podospora*.

### CR and DNP enhance fasting eNOS and Akt expression and activity

**Figure 2:**
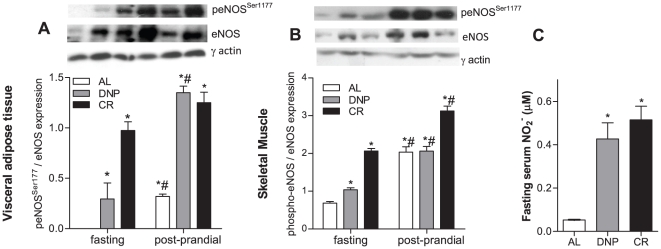
eNOS activity is increased in fasted DNP and CR tissues, and enhanced in the post-prandial period. eNOS and phospho-eNOS^Ser1177^ expression were measured in the visceral adipose tissue (A) and skeletal muscle (B) as described in Materials and Methods. Typical blots are shown above average densitometry results. Plasma nitrite (NO_2_^−^) levels (C) were measured using an NO^•^ analyzer. AL, *ad libitum*; DNP, dinitrophenol-treated mice; CR, calorically-restricted mice. *p<0.05 vs. fasting AL. ^#^p<0.05 vs. group-matched fasting.

Mitochondrial biogenesis has been shown to be dependent on NO^•^ generated by eNOS in different cells and tissues [8]; [39]. We found that during fasting, tissues from DNP and CR mice presented striking increments in both the expression and phosphorylation of eNOS (Fig. 2A–B). In the post-prandial state, eNOS activity and expression from AL tissues was enhanced, reaching levels more similar to DNP and CR animals. Enhanced fasting eNOS activity can account for mitochondrial biogenesis verified in DNP and CR groups. Indeed, we found that levels of nitrite, a general marker of the NO^•^ pool [34], was prominently increased in the serum of fasted CR and DNP animals (Fig. 2E). Nitrite levels cannot be accurately measured in post-prandial serum due to dietary contaminants.

The finding that eNOS quantity and phosphorylation increased specifically in fasted CR and DNP animals suggests that these changes involve signaling pathways sensitive to nutritional status. In fact, we have previously shown that DNP mice, similarly to CR mice, present lower titers of circulating glucose and insulin [4]. These serological alterations are commonly associated with enhanced peripheral insulin sensitivity [40]–[43]. Indeed, CR has been widely associated with improved responses to insulin [40]; [41]; [44]; [45]. Furthermore, insulin has been demonstrated to activate eNOS in a manner dependent on Akt phosphorylation [46].

**Figure 3:**
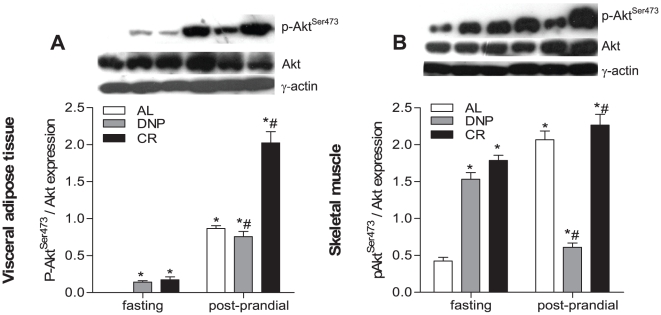
Akt expression and activity is increased in fasted DNP and CR tissues and enhanced in the post-prandial period. Akt and phospho-Akt^Ser473^ expression were measured in the visceral adipose tissue (A) and skeletal muscle (B) as described in Materials and Methods. Typical blots are shown above average densitometry results. AL, *ad libitum*; DNP, dinitrophenol-treated mice; CR, calorically-restricted mice *p<0.05 vs fasting AL. ^#^p<0.05 vs. group-matched fasting.

We thus hypothesized that modulations in insulin signaling could be involved in mitochondrial biogenesis observed in DNP and CR animals. In order to verify this possibility, we measured Akt expression and phosphorylation (Fig. 3). We found that DNP and CR increased Akt phosphorylation in the adipose tissue and skeletal muscle of fasted animals. As observed with eNOS, feeding increased Akt expression and activity many times in AL tissues. The expression and phosphorylation patterns of Akt thus closely mirror the changes found in eNOS expression and phosphorylation. These results are compatible with the idea that the regulation of eNOS activity is mediated by Akt phosphorylation and sensitive to energy metabolism [46]. Thus, our results suggest that while AL animals present high levels of eNOS activity only during the post-prandial period, DNP and CR animals maintain eNOS activity both when fasted and when fed, due to higher activation of Akt. Our findings are in line with previous literature which shows increased peripheral insulin sensitivity in CR [44]–[45].

Overall, we find that, similarly to CR, mild systemic uncoupling in mammals promoted by DNP leads to a stimulated response of Akt pathways, NO^•^ generation and mitochondrial biogenesis. These results bring further support to the concept that variations in energy metabolism, in independent ways, can converge to pathways that are determinant for lifespan extension.
